# Ivabradine in patients with acute ST-elevation myocardial infarction: a meta-analysis of randomized controlled trials

**DOI:** 10.1186/s43044-023-00351-8

**Published:** 2023-04-06

**Authors:** Bryan Richard Sasmita, Siyuan Xie, Gang Liu, Yuansong Zhu, Suxin Luo, Bi Huang

**Affiliations:** Department of Cardiology, The First Affiliated Hospital of Chongqing Medical University, Chongqing, 400016 China

**Keywords:** Acute ST-elevation myocardial infarction, Ivabradine, Heart rate, LV remodeling, LV function

## Abstract

**Background:**

Elevated resting heart rate (HR) predicts poor outcomes in patients with coronary artery disease. Ivabradine has been recommended as a second-line anti-anginal agent in chronic coronary syndrome, while there are no clear indications for acute ST-elevation myocardial infarction (STEMI).

**Results:**

We systematically searched PubMed, Medline, EMBASE, Clinical Trials.gov, and the Cochrane Central Register of Controlled Trials with search terms Ivabradine and Acute myocardial infarction. There are two study outcomes from this study: therapeutic and safety effects. Therapeutic effects include the efficacy of Ivabradine on HR, all-cause mortality, heart failure incidence, left ventricular function and remodeling. Safety effects include troponin levels and ischemic events (recurrent angina pectoris). A total of 6 RCTs was included and showed that Ivabradine was associated with greater resting HR reduction [MD − 5.40; 95%CI − 8.60, − 2.20], improvement of left ventricular ejection fraction [MD 2.98; 95%CI 0.44, 5.51], and left ventricular end systolic volume [MD − 3.81; 95%CI − 6.88, − 0.75]. However, Ivabradine had no impact on all-cause mortality [OR 0.76; 95%CI 0.35, 1.67], heart failure incidence [OR 0.61; 95%CI 0.21, 1.80], and recurrent angina pectoris [OR 0.71; 95%CI 0.50, 1.00].

**Conclusions:**

Ivabradine is safe and effective for resting HR reduction in patients with STEMI; however, it has no significant influence on mortality. These results suggest that an elevated HR is only a marker of risk but not a modifiable determinant of outcomes in patients who have suffered an acute myocardial infarction.

**Supplementary Information:**

The online version contains supplementary material available at 10.1186/s43044-023-00351-8.

## Background

The current guideline recommends a target heart rate (HR) of 50–60 beats per minute (bpm) for patients with unstable angina [1]; however, there is no clear guidance on target HR in patients with acute ST-elevation myocardial infarction (STEMI). Several studies have demonstrated that higher HR after acute myocardial infarction (AMI) is associated with increased mortality, indicating that HR control could benefit patients with AMI [2, 3].

Beta-blockers' effect on reducing the risk of re-infarction and long-term all-cause mortality remained the cornerstone therapy for HR control in patients with AMI [4]. In contrast, Ivabradine is not only recommended as a second-line HR control in patients with left ventricular ejection fraction (LVEF) ≤ 40% but is also known as a second-line anti-anginal agent in chronic coronary syndrome (CCS) [5]. Theoretically, Ivabradine could have a positive impact on patients with STEMI due to its HR control and anti-anginal effect; however, according to the European Medicine Agency [6], STEMI is one of the contraindications for Ivabradine and RCT studies aimed to evaluate Ivabradine in patients with STEMI were limited [8–13]. Therefore, we designed this meta-analysis to evaluate the efficacy and safety of Ivabradine in patients with STEMI.

## Methods

This study was conducted per standard article publication in Medical Journals, as this article has been made in coherence with the Preferred Reporting Items for Meta-Analysis PRISMA Checklist [7].

**Fig. 1:**
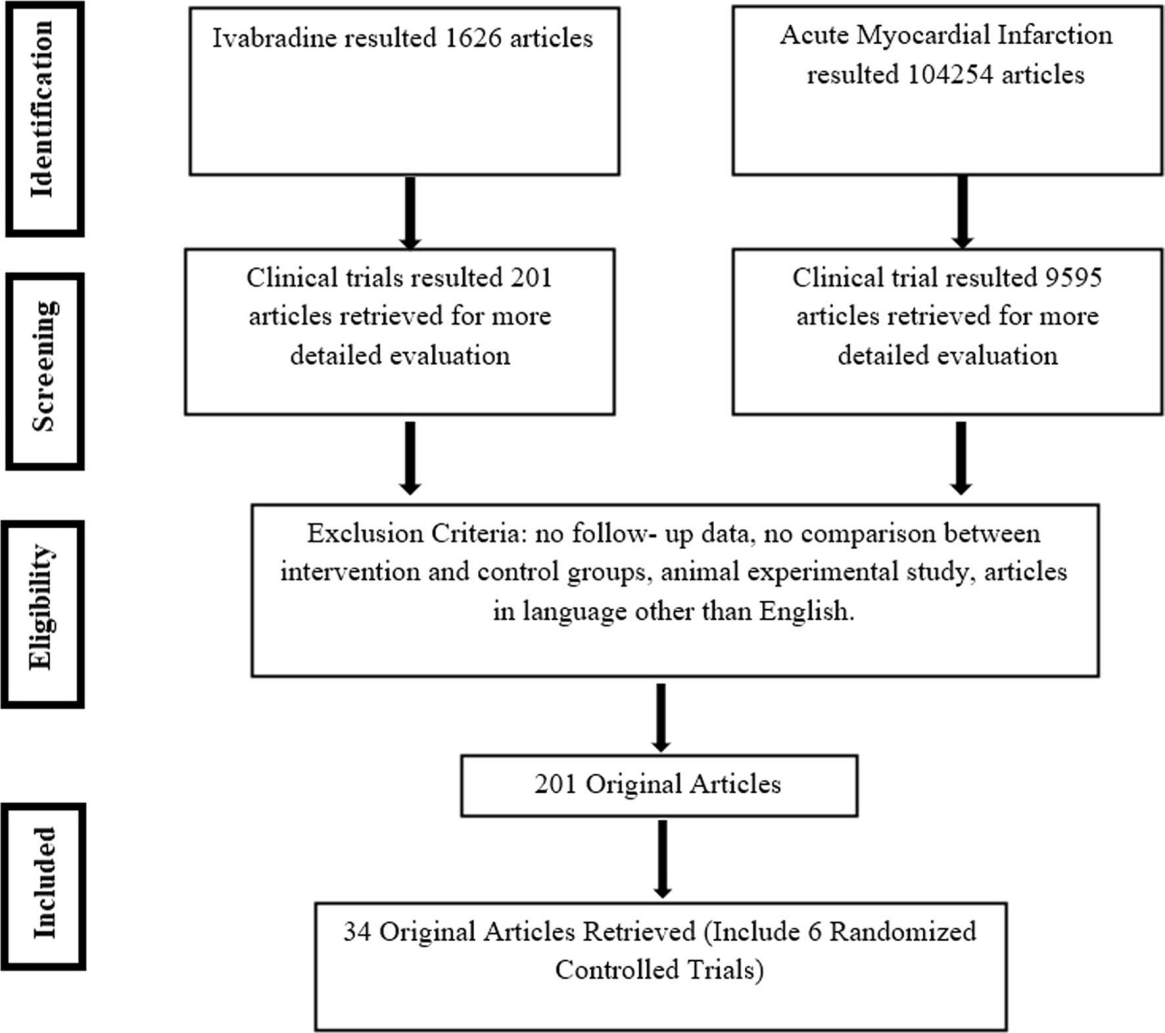
Flow diagram of data collection

Two authors (B.R.S, S.Y.X) systematically searched PubMed, Medline, EMBASE, Clinical Trials.gov, and the Cochrane Central Register of Controlled Trials with the search terms Ivabradine and AMI (Fig. 1).

Clinical trials with the following inclusion criteria were included: [1] Ivabradine used in STEMI patients and published in English; [2] Effect and safety of Ivabradine compared to non-Ivabradine group, with or without standard optimal medical treatment, including beta-blockers, antithrombotic agents, lipid-lowering agents, nitrates, renin-angiotensin system (RAS) inhibitors; [3] Pre- and post-treatment echocardiographic assessment (Additional file 1: Table S1). The exclusion criteria were as follows: (1) Non-human studies; (2) Articles in a language other than English; (3) No follow-up data; (4) Non-AMI, including old myocardial infarction and CCS; (4) No comparison between intervention and control groups (Table 1).

**Table 1:** Characteristics of included studies

Trial	Types of Study	Population	N	Ivabradine regimen	Non-Ivabradine Group	Endpoint	Time (days)
Priti, et al. [8]	RCT	Acute Inferior wall STEMI	464	2.5–7.5 mg bid, PO	Metoprolol	MACE	30
Barilla, et al. [9]	RCT	CS complicating STEMI	58	2.5–7.5 mg bid, PO/NGT	Standard treatment	NT-proBNP	180
Rezq, et al. [10]	RCT	Acute Anterior wall STEMI	670	5 mg bid, PO	Bisoprolol	MACE	365
Fasullo, et al. [11]	RCT	Acute Anterior wall STEMI	155	2.5–7.5 mg bid, PO	Metoprolol Succinate	LV remodeling	60
Steg, et al. [12]	RCT	Acute STEMI	124	5 mg, IV	Placebo	Heart Rate	120
Xu, et al. [13]	RCT	Acute STEMI	66	2.5–7.5 mg bid, PO	Metoprolol	LV remodeling	180

*RCT* randomized controlled trial; *STEMI* ST Elevation myocardial infarction; *CS* cardiogenic shock; *MACE* major adverse cardiovascular events; *LV* left ventricular; *NT-proBNP* N-terminal-pro hormone brain natriuretic peptide

Therapeutic effects included changes in HR, left ventricular ejection fraction, left-ventricular end-diastolic volume (LVEDV), left ventricular end-systolic volume (LVESV), all-cause mortality, and heart failure incidence. Safety effects included troponin levels and ischemic events (recurrent angina pectoris).

Data analysis was done by using RevMan 5.4. Dichotomous data were reported by using Mantel–Haenszel statistical method, fixed/random effects analysis model, and odds ratio (OR) effect measure with 95% CIs. In addition, continuous variables were evaluated using mean differences (MD) with 95% CIs. The effect model was preferred in data analysis depending on the degree of heterogeneity and P-value, a fixed-effect model was used if I^2^ < 50% and P-value > 0.10, while the random effect model was preferred in the high heterogeneity I^2^ > 50% and low P-value < 0.10. If heterogeneity was detected, subgroup analyses to explore the source of heterogeneity were conducted. Meanwhile, a sensitivity analysis to evaluate the robustness of the outcomes was done by removing the study with a high risk of selection bias (random sequence generation or allocation concealment).

The Cochrane risk of bias domains was used to analyze the bias ratings of each study. The selection of domains included random sequence generation, allocation concealment, blinding of participants and personnel, blinding of outcome assessment, incomplete outcome data, selective reporting, and other bias. Ratings of bias were divided into low risk, unclear risk, and high risk. Quality of evidence extracted by two independent investigators (B.R.S and Y.S.Z) and the disagreement about inclusion data was evaluated by a third investigator (B.H) through a discussion and consensus.

## Results

A total of 1537 participants (790 in the Ivabradine group and 747 in the non-Ivabradine group) were enrolled in the present meta-analysis [8–13]. The outcomes were the safety and efficacy of Ivabradine compared to the non-Ivabradine group in STEMI patients. The high heterogeneity was attributed to a distinct measurement index, insufficient studies on preferred outcomes, and different baseline characteristics of included studies, such as sample size, age, gender, and follow-up time (Additional file 1: Table S2).

**Fig. 2:**
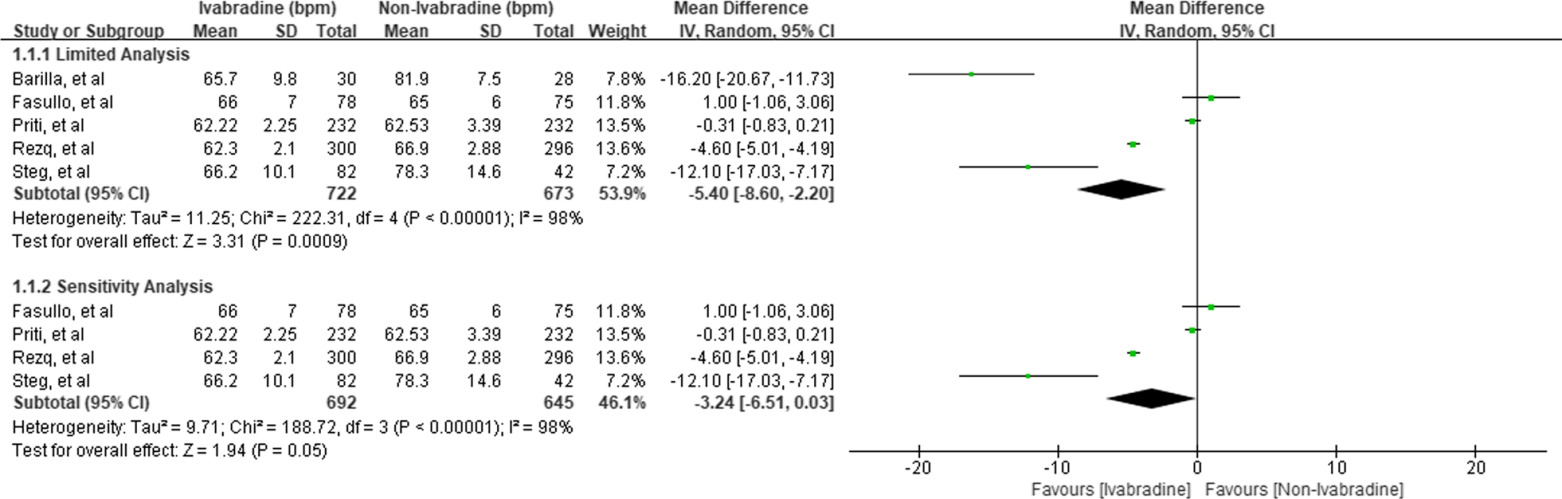
Effect of Ivabradine on Heart Rate reduction

Five RCTs included HR change as one of the outcomes [8–12] (Fig. 2) and showed Ivabradine was associated with lower resting HR compared to the non-Ivabradine group [MD − 5.40; 95%CI − 8.60, − 2.20]. Furthermore, sensitivity analysis with the removal of high-risk selection bias study showed inconsistent findings on the effect of Ivabradine for HR reduction [MD − 3.24; 95%CI − 6.51, 0.03].

**Fig. 3:**
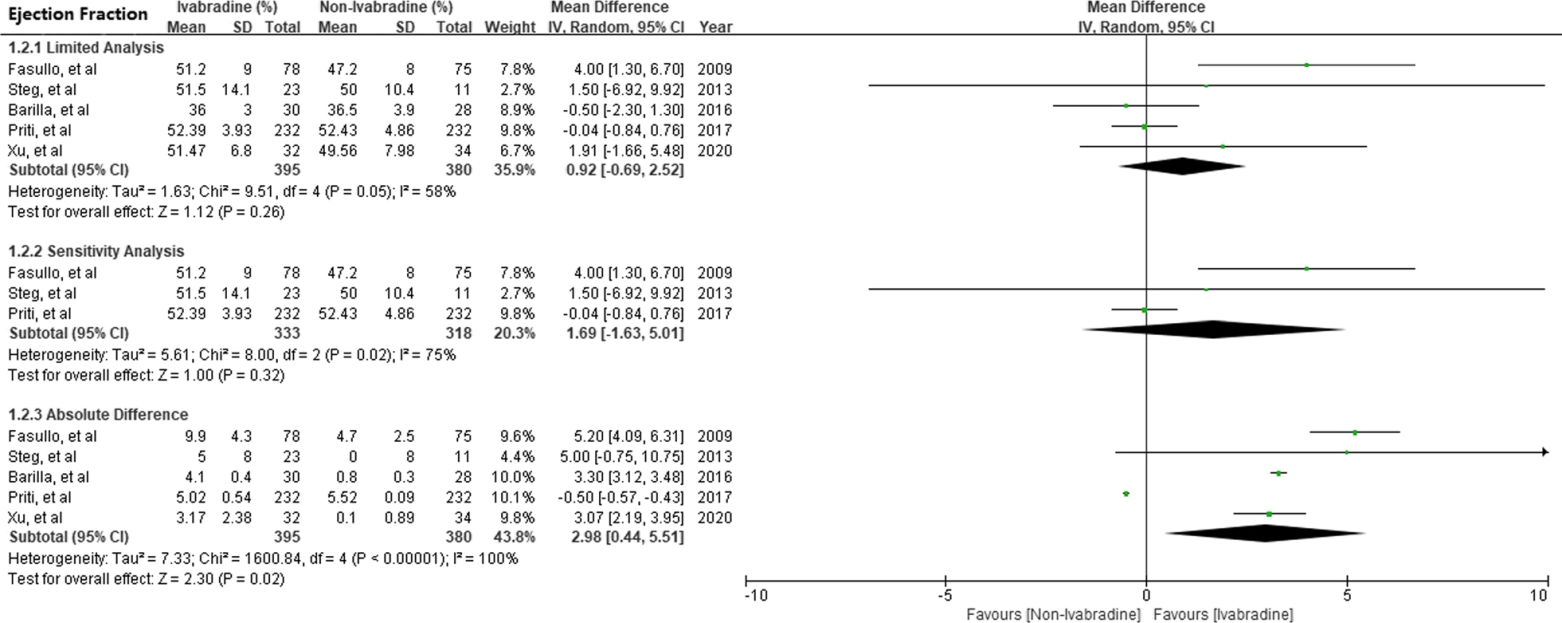
Effect of Ivabradine compared with non-Ivabradine group in LV ejection fraction

In the present meta-analysis, 5 RCTs with 775 patients evaluated the effect of Ivabradine on LVEF improvement [8, 9, 11–13]. Limited analysis [MD 0.92; 95%CI -0.69, 2.52] and sensitivity analysis [MD 1.69; 95%CI − 1.63, 5.01] showed that Ivabradine had no significant effect in improving LVEF in patients with STEMI. However, due to the different baseline LVEF in each study, the absolute differences analysis was further adopted, and it showed that Ivabradine was associated with LVEF improvement [MD 2.98; 95%CI 0.44, 5.51] (Fig. 3).

**Fig. 4:**
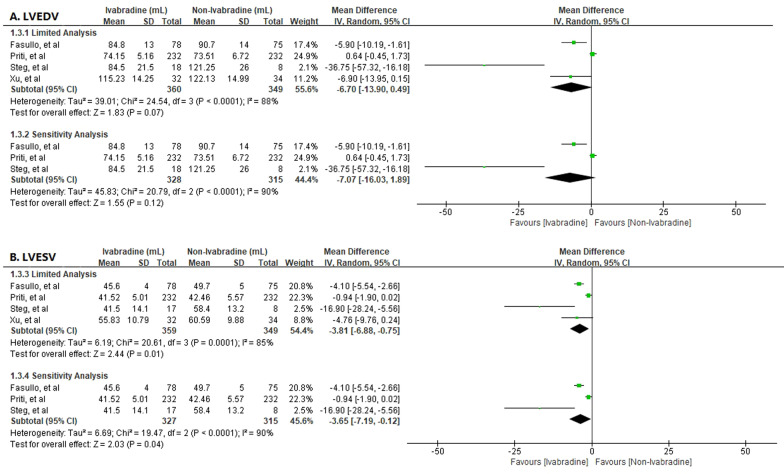
Effect of Ivabradine on LV remodeling. A. Left ventricular end diastolic volume (LVEDV); B. Left ventricular end systolic volume (LVESV)

The present meta-analysis assessed the impact of Ivabradine on LVESV and LVEDV [8, 11–13] and showed that Ivabradine improved LVESV [MD − 3.81; 95%CI − 6.88, − 0.75] but had no significant effect on LVEDV [MD − 6.70; 95%CI − 13.90, 0.49]. Furthermore, sensitivity analysis revealed that Ivabradine maintained its positive effect on LVESV [MD − 3.65; 95%CI − 7.19, − 0.12] while had no effect on LVEDV [MD − 7.07; 95%CI − 16.03, 1.89] (Fig. 4).

**Fig. 5:**
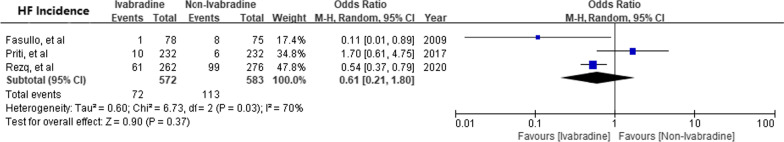
Effect of Ivabradine on Heart Failure Incidence

Three RCTs evaluated the impact of Ivabradine on heart failure incidence [8, 10, 11] and showed that Ivabradine was not associated with reduced risk of heart failure incidence [OR 0.61; 95%CI 0.21, 1.80] (Fig. 5).

**Fig. 6:**
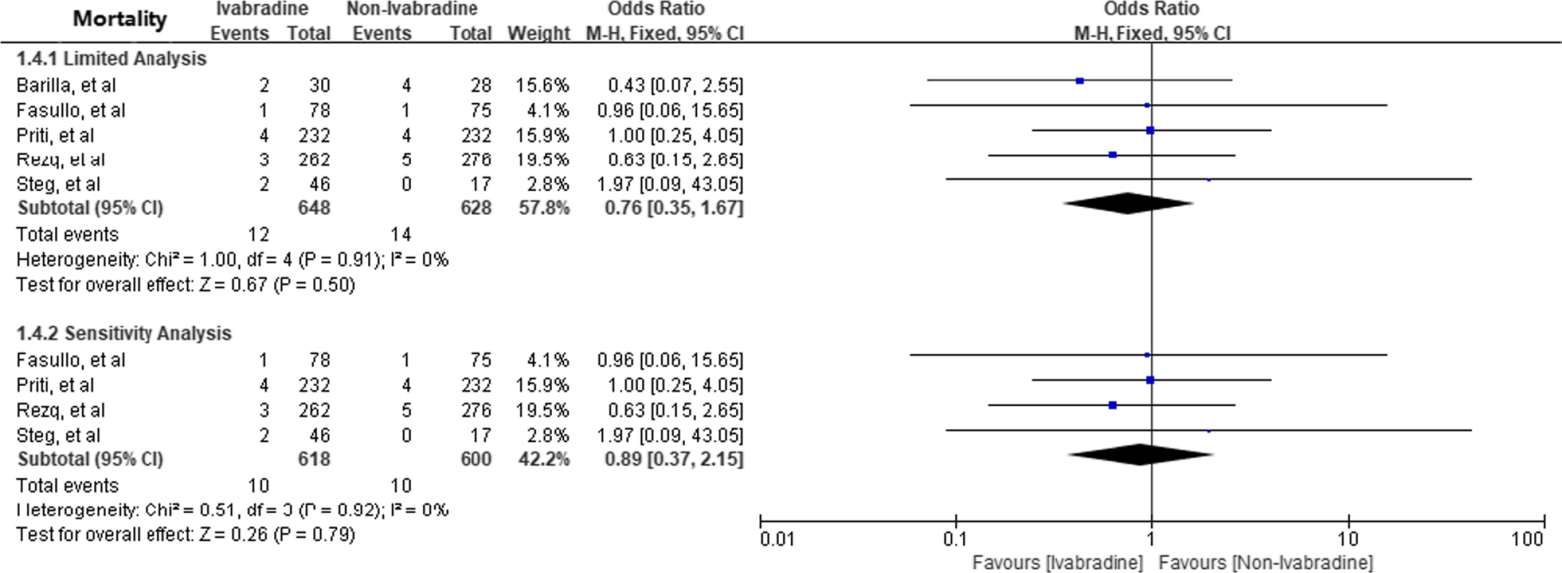
Effect of Ivabradine on All-cause mortality

Five RCTs (648 patients in Ivabradine group and 628 patients in non-ivabradine group) were included to assess the impact of Ivabradine on all-cause mortality [8–12] (Fig. 6). Limited analysis [OR 0.76; 95%CI 0.35, 1.67] and sensitivity analysis [OR 0.89; 95%CI 0.37, 2.15] revealed that Ivabradine had no significant effect on all-cause mortality.

**Fig. 7:**
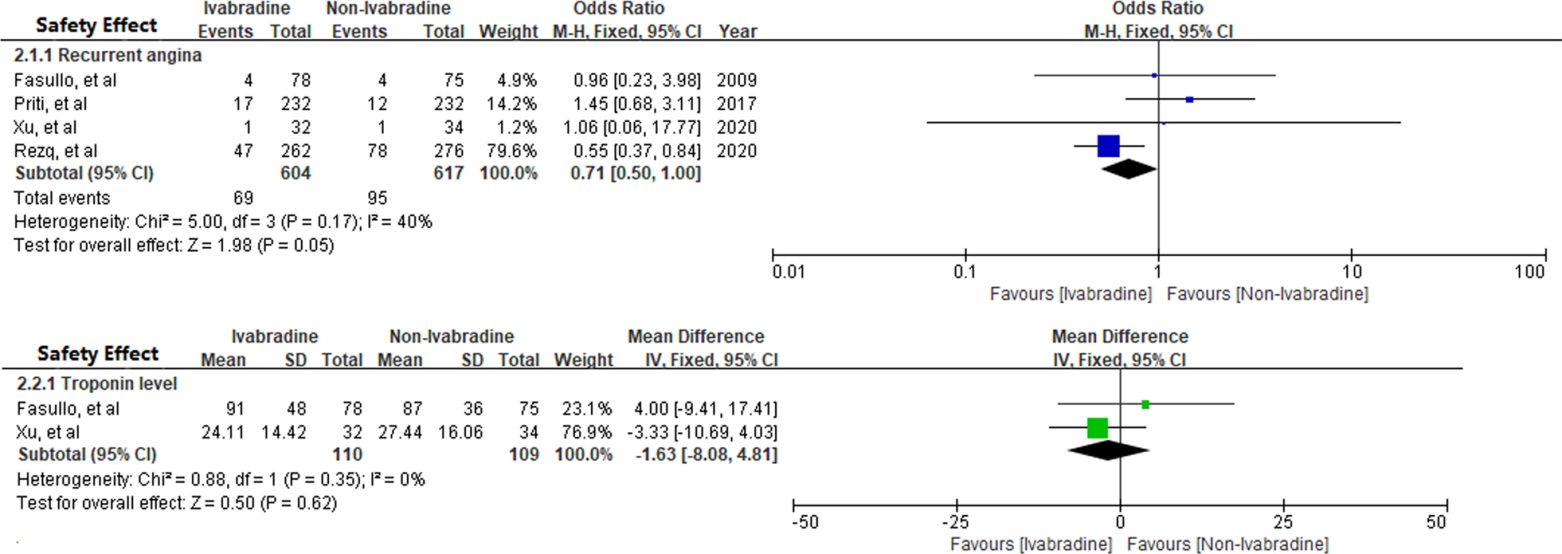
The safety effect of Ivabradine

Four RCTs assessed the safety of Ivabradine in patients with STEMI [8, 10, 11, 13] and demonstrated Ivabradine had no effects on recurrent angina pectoris [8, 10, 11] [OR 0.71; 95%CI 0.50, 1.00] and cardiac troponin reduction [11, 13] [MD − 1.63; 95%CI − 8.08, 4.81] (Fig. 7).

**Fig. 8:**
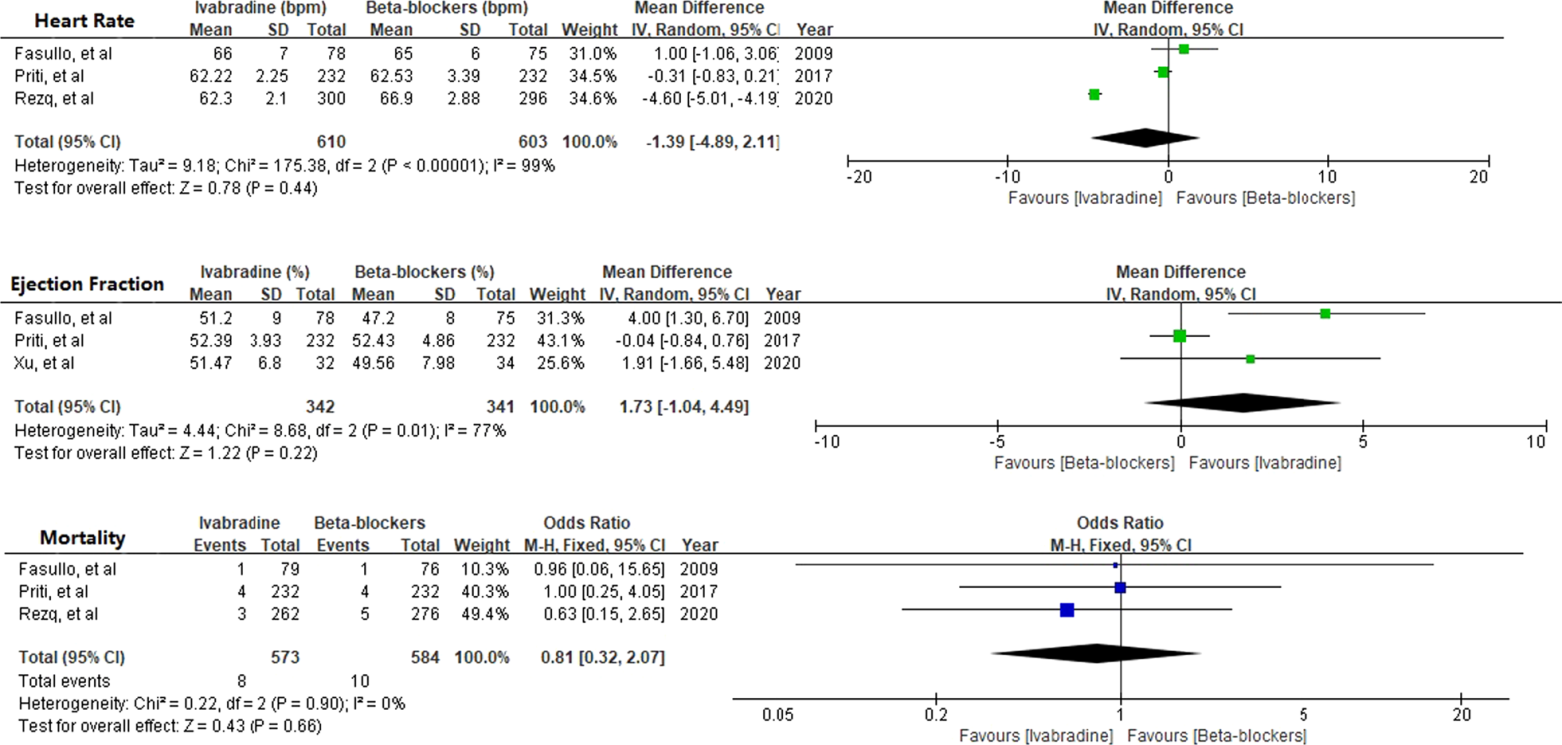
Comparison of the safety and efficacy of Ivabradine vs. Beta-blockers

The present study also compared Ivabradine and beta-blockers for HR control, LVEF improvement, and mortality. However, we found that Ivabradine was not superior to beta-blockers in terms of HR reduction [MD − 1.39; 95%CI − 4.89, 2.11], LVEF improvement [MD 1.73, 95%CI − 1.04, 4.49], and mortality [OR 0.81; 95%CI 0.32, 2.07] (Fig. 8).

**Fig. 9:**
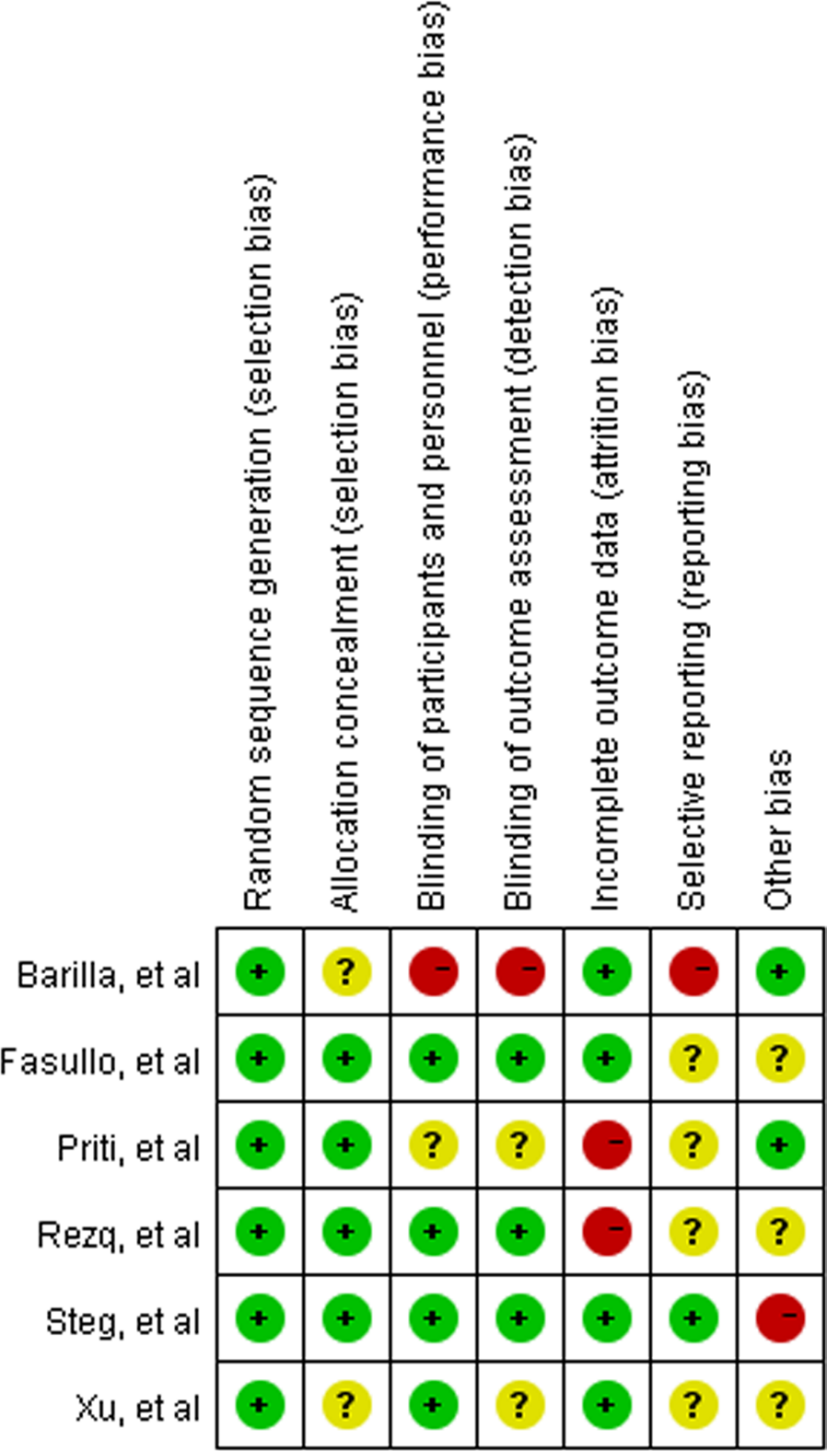
Risk of bias

Based on the Cochrane Collaboration for risk of bias assessment criteria, enrolled studies presented with various risks of bias (Fig. 9). Moreover, the assessment of other possible biases is uncertain due to insufficient information from these studies.

High heterogeneity investigation in the resting HR and LVEF was stratified based on the follow-up duration. Follow-up duration was divided into two groups, ≤ 120 days [8, 11, 12] and > 120 days [9, 10, 13]. In terms of HR reduction, both groups had a high heterogeneity [MD − 2.78; 95%CI − 6.83, 1.27; I^2^ 91% for ≤ 120 days and MD − 10.18; 95%CI − 21.54, 1.18; I^2^ 96% for > 120 days, respectively]. However, a longer treatment seemed to be associated with higher resting HR reduction, although the difference was not statistically significant. As for LVEF changes, a longer treatment had a significantly lower heterogeneity [MD − 0.01; 95%CI − 1.62, 1.60, I^2^ 28% vs. MD 1.69; 95%CI − 1.63, 5.01, I^2^ 75%, respectively], indicating follow-up duration may contribute to the heterogeneity of LVEF changes.

## Discussion

The present meta-analysis demonstrated that Ivabradine treatment was effective for HR reduction and could improve LV function and cardiac remodeling. To the best of our knowledge, this is the first RCT-based meta-analysis to evaluate the effect of Ivabradine in STEMI patients.

The relationship between elevated HR and myocardial ischemia has long been established. The Framingham study found a significant association between a higher HR with coronary heart disease and sudden coronary death [14, 15]. The pathophysiology of myocardial ischemia was traditionally attributed to the imbalance between the oxygen supply and demand of the myocardium. Increased HR can reduce the myocardial blood supply, increase myocardial energy/oxygen demand, and shorten the diastolic duration [16, 17]. This theory was supported by a BEAUTIFUL study [18], where a reduction in HR was associated with a reduced incidence of coronary artery disease in patients with HR of 70 bpm or greater.

Beta-blocker is the primary choice for HR reduction and anti-anginal agents in patients with AMI mainly due to their effect on decreasing myocardial oxygen demand, improving left ventricular hemodynamic function, and altering cardiac remodeling [19, 20]. In addition, according to the current guidelines, long-term treatment with beta-blocker is recommended for all AMI patients without contraindications [21, 22]. Although beta-blocker is considered a cornerstone therapy in AMI patients, several contraindications and side effects, including hypotension, peripheral vascular disease, worsening cardiac function, hemodynamic instability, asthma, and acute exacerbation of chronic obstructive pulmonary disease limit its use in clinical practice [23–25].

Ivabradine is a new pure bradycardic agent without affecting cardiac conductivity. Ivabradine is a selective inhibitory of cardiac pacemaker cell channels to lower resting HR through inhibition of If channel in the sinus node, thus prolonging diastolic depolarization of a pacemaker action potential [26]. In terms of efficacy, the present meta-analysis demonstrated that Ivabradine treatment was associated with significant resting HR reduction. This result was anticipated, as SIGNIFY study [27] has confirmed our findings in patients with stable coronary artery disease. Meanwhile, our study demonstrated that adding Ivabradine to standard background therapy did not improve mortality, recurrent angina pectoris, and heart failure incidence.

Further data from SIGNIFY study [27] has shown that Ivabradine not only caused HR reduction but also reduced afterload, therefore we try to assess the possibility of Ivabradine in left ventricular function. At first, our statistical analysis did not find the beneficial effect of Ivabradine on LVEF improvement. However, after comparing the two groups' absolute differences in LVEF, we found that Ivabradine led to the preservation of LV function compared to the non-Ivabradine group. In fact, there is another meta-analysis conducted by Wang et al. [28], who found that Ivabradine had higher LVEF improvement [MD 3.17; 95%CI 2.12, 4.23] compared to our study [MD 2.98; 95%CI 0.44, 5.51]. The possible explanation was mainly due to the differences in the search strategy and inclusion criteria, as our study did not include studies published in Chinese. Furthermore, although several meta-analyses conducted by Chen et al. [29], Maagard et al. [30], and Kang et al. [31] had a similar inclusion as ours, we focused on pure acute STEMI patients and our results could provide efficacy and safety issues for Ivabradine in patients with STEMI. Consistent with previous findings, our meta-analysis also found Ivabradine was not associated with improved outcome, suggesting that an elevated HR was not a modifiable determinant of outcomes in patients with STEMI.

Several hypotheses were proposed in regards to the potential mechanisms of Ivabradine for improving cardiac remodeling, such as improvement of endothelial function, a reversal in electrophysiologic changes, modification of cardiac myocyte function, reduction of renin–angiotensin–aldosterone system stimulation and sympathetic drive [32]. Therefore, we try to assess the possibility of Ivabradine to improve left ventricular remodeling in AMI patients by analyzing the changes in LVEDV and LVESV, as several studies have confirmed the prognostic value of LVEDV and LVESV in AMI patients [33, 34]. Through limited and sensitivity analysis, we found that Ivabradine treatment could improve LVESV but not LVEDV. The precise mechanism was not well understood; however, it may be associated with the dynamic changes of HR and LVEF. To date, there is a lack of studies specifically aimed at evaluating the effect of Ivabradine on left ventricular remodeling in AMI patients, and whether it can improve cardiac remodeling deserves further investigation.

Based on our subgroup analysis, we found that the longer treatment duration (> 120 days) did not affect LVEF change; however, it had a more potent effect on HR reduction in comparison to the shorter treatment group (< 120 days). In addition, a recent clinical trial by Shen et al. [4] found that HR of more than 78 bpm was independently associated with an increased risk of long-term all-cause mortality in patients with STEMI. Therefore, long-term use of Ivabradine provided beneficial effects such as HR control, reduced oxygen consumption, and improved LVEF in patients with STEMI.

Ivabradine is recommended as a second-line anti-anginal agent in patients with CCS [5]; however, according to the European Medicine Agency [6], the use of Ivabradine for unstable angina and AMI is contraindicated. The main reason is that the preliminary results of SIGNIFY trial showed a small but statistically significant increase in the combined risk of cardiovascular death and non-fatal MI with Ivabradine compared with placebo [6, 27]. Meanwhile, our present meta-analysis demonstrated that Ivabradine was safe and effective in patients with STEMI, indicating Ivabradine is an option for patients with STEMI, especially those with contraindications for beta-blocker.

There are several limitations to this meta-analysis. First, some of the included studies have a small sample size, short follow-up time, and different outcomes, limiting the statistical power and preferred outcomes. Second, included studies only provided average doses of Ivabradine and we could not get the dose–effect relationship. Therefore, more large-scale studies are still needed to elucidate the efficacy and safety of Ivabradine in AMI patients.

## Conclusions

Ivabradine is safe and effective for resting HR reduction in patients with STEMI; however, it has no significant influence on mortality. These results suggest that an elevated HR is only a marker of risk but not a modifiable determinant of outcomes in patients who have suffered an AMI.

## Supplementary Information

**Additional file 1.** **Supplement Table 1.** Baseline Characteristics of Included Studies. **Supplement Table 2.** Post-Follow up Outcomes.

## Data Availability

All data relevant to the study are included in the article or uploaded as Additional files. Data can also be requested from the corresponding author.

## Abbreviations

HR: Heart rate
STEMI: ST-elevation myocardial infarction
AMI: Acute myocardial infarction
Bpm: Beats per minute
LVEF: Left ventricular ejection fraction
CCS: Chronic coronary syndrome
LVEDV: Left ventricular end-diastolic volume
LVESV: Left ventricular end-systolic volume
RCT: Randomized controlled trial
MD: Mean differences
OR: Odds ratio

## References

[1] Anderson JL, Adams CD, Antman EM (2007). ACC/AHA 2007 guidelines for the management of patients with unstable angina/non-ST-Elevation myocardial infarction: a report of the American College of Cardiology/American Heart Association Task Force on Practice Guidelines (Writing Committee to Revise the 2002 Guidelines for the Management of Patients With Unstable Angina/Non-ST-Elevation Myocardial Infarction) developed in collaboration with the American College of Emergency Physicians, the Society for Cardiovascular Angiography and Interventions, and the Society of Thoracic Surgeons endorsed by the American Association of Cardiovascular and Pulmonary Rehabilitation and the Society for Academic Emergency Medicine. J Am Coll Cardiol.

[2] Jabre P, Roger VL, Weston SA (2014). Resting heart rate in first year survivors of myocardial infarction and long-term mortality: a community study. Mayo Clin Proc.

[3] Shen J, Liu G, Yang Y (2021). Prognostic impact of mean heart rate by Holter monitoring on long-term outcome in patients with ST-segment elevation myocardial infarction undergoing percutaneous coronary intervention. Clin Res Cardiol.

[4] Safi S, Sethi NJ, Nielsen EE, Feinberg J, Jakobsen JC, Gluud C (2019). Beta-blockers for suspected or diagnosed acute myocardial infarction. Cochrane Database Syst Rev.

[5] Knuuti J, Wijns W, Saraste A (2020). 2019 ESC Guidelines for the diagnosis and management of chronic coronary syndromes. Eur Heart J.

[6] Drug Safety Update (2014) 7(11): S1

[7] Liberati A, Altman DG, Tetzlaff J (2009). The PRISMA statement for reporting systematic reviews and meta-analyses of studies that evaluate healthcare interventions: explanation and elaboration. BMJ.

[8] Priti K, Ranwa BL, Gokhroo RK, Kishore K, Bisht DS, Gupta S (2017). Ivabradine vs metoprolol in patients with acute inferior wall myocardial infarction-"Expanding arena for ivabradine". Cardiovasc Ther.

[9] Barilla F, Pannarale G, Torromeo C (2016). Ivabradine in patients with ST-elevation myocardial infarction complicated by cardiogenic shock: a preliminary randomized prospective study. Clin Drug Investig.

[10] Rezq A, Saad M, Al Mahmoudy A, El Nozahi M (2020). Value of Ivabradine in patients with anterior ST-Elevation myocardial infarction: the VIVA-STEMI study. Cardiol Cardiovasc Med.

[11] Fasullo S, Cannizzaro S, Maringhini G (2009). Comparison of ivabradine versus metoprolol in early phases of reperfused anterior myocardial infarction with impaired left ventricular function: preliminary findings. J Card Fail.

[12] Steg P, Lopez-de-Sà E, Schiele F (2013). Safety of intravenous ivabradine in acute ST-segment elevation myocardial infarction patients treated with primary percutaneous coronary intervention: a randomized, placebo-controlled, double-blind, pilot study. Eur Heart J Acute Cardiovasc Care.

[13] Xu Y, Zhang W, Zhong X (2021). Effect of early use of ivabradine on left ventricular remodeling after primary percutaneous coronary intervention in patients with acute ST-segment elevation myocardial infarction: a pilot test. Ann Noninvasive Electrocardiol.

[14] Kannel WB, Kannel C, Paffenbarger RS, Cupples LA (1987). Heart rate and cardiovascular mortality: the Framingham Study. Am Heart J.

[15] Ho JE, Larson MG, Ghorbani A (2014). Long-term cardiovascular risks associated with an elevated heart rate: the Framingham Heart Study. J Am Heart Assoc.

[16] Heusch G, Schulz R (2007). The role of heart rate and the benefits of heart rate reduction in acute myocardial ischaemia. Eur Heart J Suppl.

[17] Hoffman JI, Buckberg GD (2014). The myocardial oxygen supply:demand index revisited. J Am Heart Assoc.

[18] Fox K, Ford I, Steg PG, Tendera M, Ferrari R, Investigators B (2008). Ivabradine for patients with stable coronary artery disease and left-ventricular systolic dysfunction (BEAUTIFUL): a randomised, double-blind, placebo-controlled trial. Lancet.

[19] Herlitz J, Elmfeldt D, Hjalmarson A (1983). Effect of metoprolol on indirect signs of the size and severity of acute myocardial infarction. Am J Cardiol.

[20] Hu K, Gaudron P, Ertl G (1998). Long-term effects of beta-adrenergic blocking agent treatment on hemodynamic function and left ventricular remodeling in rats with experimental myocardial infarction: importance of timing of treatment and infarct size. J Am Coll Cardiol.

[21] Amsterdam EA, Wenger NK, Brindis RG (2014). 2014 AHA/ACC guideline for the management of patients with non-ST-elevation acute coronary syndromes: a report of the american college of cardiology/american heart association task force on practice guidelines. J Am Coll Cardiol.

[22] O'Gara PT, Kushner FG, Ascheim DD (2013). 2013 ACCF/AHA guideline for the management of ST-elevation myocardial infarction: a report of the American college of cardiology foundation/American heart association task force on practice guidelines. Circulation.

[23] Wiysonge CS, Bradley HA, Volmink J, Mayosi BM, Opie LH (2017). Beta-blockers for hypertension. Cochrane Database Syst Rev.

[24] Beumer HM (1974). Adverse effects of beta-adrenergic receptor blocking drugs on respiratory function. Drugs.

[25] Frohlich ED, Tarazi RC, Dustan HP (1969). Peripheral arterial insufficiency. A complication of beta-adrenergic blocking therapy. JAMA.

[26] DiFrancesco D, Camm JA (2004). Heart rate lowering by specific and selective I(f) current inhibition with ivabradine: a new therapeutic perspective in cardiovascular disease. Drugs.

[27] Fox K, Ford I, Ferrari R (2014). Ivabradine in stable coronary artery disease. N Engl J Med.

[28] Wang B, Zhang X, Chen J (2021). Effectiveness and safety of ivabradine in the treatment of acute myocardial infarction: a systematic review and meta-analysis. Ann Palliat Med.

[29] Chen A, Elia N, Dunaiceva J, Rudiger A, Walder B, Bollen PB (2020). Effect of ivabradine on major adverse cardiovascular events and mortality in critically ill patients: a systematic review and meta-analyses of randomised controlled trials with trial sequential analyses. Br J Anaesth.

[30] Maagaard M, Nielsen EE, Sethi NJ (2020). Effects of adding ivabradine to usual care in patients with angina pectoris: a systematic review of randomised clinical trials with meta-analysis and Trial Sequential Analysis. Open Heart..

[31] Kang S, Li CJ, Zhang XM (2017). Ivabradine has a neutral effect on mortality in randomized controlled trials. Medicine.

[32] Vercauteren M, Favre J, Mulder P, Mahlberg-Gaudin F, Thuillez C, Richard V (2007). Protection of endothelial function by long term heart rate reduction induced by ivabradine in a rat model of chronic heart failure. Fundam Clin Pharmacol.

[33] White HD, Norris RM, Brown MA, Brandt PW, Whitlock RM, Wild CJ (1987). Left ventricular end-systolic volume as the major determinant of survival after recovery from myocardial infarction. Circulation.

[34] Reindl M, Reinstadler SJ, Tiller C (2019). Prognosis-based definition of left ventricular remodeling after ST-elevation myocardial infarction. Eur Radiol.

