# Supplementary material for: Ivabradine in patients with acute ST-elevation myocardial infarction: a meta-analysis of randomized controlled trials
Source: Egypt Heart J. 2023 Apr 6;75:25. doi: 10.1186/s43044-023-00351-8 (PMC10079792; doi:10.1186/s43044-023-00351-8)
Supplement: Supplementary file 1 — Additional file 1. Supplement Table 1. Baseline Characteristics of Included Studies. Supplement Table 2. Post-Follow up Outcomes. [file 43044_2023_351_MOESM1_ESM.docx]

Supplement Table 1. Baseline Characteristics of Included Studies

| **Studies** | **Priti, et al ^[8]^** | **Barilla, et al ^[9]^** | | **Resq, et al ^[10]^** | | **Fasullo, et al ^[11]^** | **Steg, et al ^[12]^** | **Xu, et al ^[13]^** |
| --- | --- | --- | --- | --- | --- | --- | --- | --- |
| ***Study Arm***  Non-Ivabradine  Ivabradine | 232  232 | 28  30 | 335  335 | | 76  79 | | 42  82 | 34  32 |
| ***Age***  Non-Ivabradine  Ivabradine | 54.6±9.03  54.6±10.64 | 54.4±10.4  56.3±9.7 | 57.0±11.6  55.0±9.2 | | 62.1±7.5  61.6±8.3 | | 58.0±11.6  60.1±10.8 | 51.5±9.55  51.28±9.42 |
| ***Men (%)***  Non-Ivabradine  Ivabradine | 178 (75)  177 (73.81) | 18 (64.3)  21 (70) | 301 (90)  290 (86.7) | | 53 (69.6)  52 (65.8) | | 33 (79)  64 (78) | 33 (97.06)  31 (96.84) |
| ***Cardiac Parameters*** |  |  |  | |  | |  |  |
| Ejection Fraction (%)  Non-Ivabradine  Ivabradine | 46.91±4.95  47.37±3.39 | 35.7±3.6  31.9±3.4 | 49.7 ±10.6  51.6± 8.6 | | 42.5±5.5  41.3±4.7 | | 45.0  50.0 | 49.46±8.87  48.30±8.78 |
| Heart Rate (beats/min)  Non-Ivabradine  Ivabradine | 82.53± 7.36  81.70± 7.21 | 94.6±6.0  97.2±6.8 | 87.7±3.1  87.9±2.46 | | 92±7  91±6 | | 87.4±8.0  88.0±10.0 | N/A |
| Troponin I  Non-Ivabradine  Ivabradine | N/A | N/A | N/A | | N/A | | N/A | 10.66±14.47  19.57±42.23 |
| ***MRI Parameters*** |  |  |  | |  | |  |  |
| LVEDV (ml)  Non-Ivabradine  Ivabradine | 97.15±5.22  95.98±7.98 | N/A | N/A | | 89.5±14  90.5±16 | | 121.5  96.0 | 97.91±16.72  95.72±14.20 |
| LVESV (ml)  Non-Ivabradine  Ivabradine | 53.23±6.89  52.70±6.13 | N/A | N/A | | 50.1±6.3  50.7±8.0 | | 52.9  50.0 | 49.03±8.37  49.50±10.84 |
| ***Medications*** |  |  |  | |  | |  |  |
| Beta-blocker  Non-Ivabradine  Ivabradine | 232  0 | 0  0 | 335  335 | | 76  0 | | 0  0 | 34  32 |
| Statins  Non-Ivabradine  Ivabradine | N/A | N/A | N/A | | 76  79 | | 28  42 | 34  32 |
| Aspirin  Non-Ivabradine  Ivabradine | N/A | N/A | N/A | | 76  79 | | N/A | 34  32 |
| ADP-R inhibitor  Non-Ivabradine  Ivabradine | N/A | N/A | N/A | | 76  79 | | N/A | 34  32 |

Supplement Table 2. Post-Follow up Outcomes

| **Studies** | **Priti, et al ^[8]^** | **Barilla, et al ^[9]^** | **Resq, et al ^[10]^** | **Fasullo, et al ^[11]^** | **Steg, et al ^[12]^** | **Xu, et al ^[13]^** |
| --- | --- | --- | --- | --- | --- | --- |
| ***Study Arm***  Non-Ivabradine  Ivabradine | 232  232 | 28  30 | 335  335 | 76  79 | 42  82 | 34  32 |
| ***Ejection Fraction (%)***  Non-Ivabradine  Ivabradine | 52.43±4.86  52.39±3.93 | 36.5±3.9  36.0±3.0 | N/A | 47.2±8  51.2±9 | 54  53 | 49.56±7.98  51.47±6.40 |
| ***Heart Rate (bpm)***  Non-Ivabradine  Ivabradine  ***Echocardiographic Parameters*** | 62.53±3.39  62.22±2.25 | 84.1±7.3  72.2±6.1 | 66.9±2.88  62.3±2.1 | 65±6  66±7 | 78.3±14.6  66.2±10.1 | N/A |
| LVEDV (ml)  Non-Ivabradine  Ivabradine | 73.51±6.72  74.15±5.16 | 67±1  65±1 | N/A | 90.7±14  84.8±13 | 121.25±26  84.5±21.5 | 122.13±14.99  115.23±14.25 |
| LVESV (ml)  Non-Ivabradine  Ivabradine | 42.46±5.57  41.52±5.01 | N/A | N/A | 49.7±5  45.6±4 | 58.4±13.2  41.5±14.1 | 60.59±9.88  55.83±10.79 |
